# European Headache Federation (EHF) critical re-appraisal and meta-analysis of oral drugs in migraine prevention—part 2: flunarizine

**DOI:** 10.1186/s10194-023-01657-3

**Published:** 2023-09-19

**Authors:** Christina I. Deligianni, Simona Sacco, Esme Ekizoglu, Derya Uluduz, Raquel Gil-Gouveia, Antoinette MaassenVanDenBrink, Raffaele Ornello, Margarita Sanchez-del-Rio, Uwe Reuter, Jan Versijpt, Tessa de Vries, Muizz Hussain, Dena Zeraatkar, Christian Lampl

**Affiliations:** 1 Department of Neurology, Athens Naval Hospital, Athens, Greece; 2 Department of Biotechnological and Applied Clinical Sciences, University of L´Aquila, L´Aquila, Italy; 3 Department of Neurology, Istanbul University Istanbul Faculty of Medicine, Istanbul, Turkey; 4 Department of Neurology, Istanbul University-Cerrahpasa Medical Faculty, Istanbul, Turkey; 5 Neurology Department, Hospital da Luz Headache Center, Hospital da Luz Lisboa, Lisbon, Portugal; 6 Center for Interdisciplinary Research in Health, Universidade Católica Portuguesa, Lisbon, Portugal; 7 Department of Internal Medicine, Erasmus MC Medical Center, Rotterdam, The Netherlands; 8 Department of Neurology, Clinica Universidad de Navarra, Madrid, Spain; 9 Department of Neurology, Charité Universitätsmedizin Berlin, Berlin, Germany and Universitätsmedizin Greifswald, Greifswald, Germany; 10 Department of Neurology, Vrije Universiteit Brussel (VUB), Universitair Ziekenhuis Brussel (UZ Brussel), Brussels, Belgium; 11 Department of Anesthesia and Department of Health Research Methods, Evidence and Impact, McMaster University, Hamilton, Canada; 12 Department of Neurology and Stroke Unit, Konventhospital Barmherzige Brüder Linz, Linz, Austria; 13 Headache Medical Center Linz, Linz, Austria

**Keywords:** Migraine, Prophylactic treatment, Flunarizine, Meta-analysis

## Abstract

**Objective:**

Novel disease-specific and mechanism-based treatments sharing good evidence of efficacy for migraine have been recently marketed. However, reimbursement by insurers depends on treatment failure with classic anti-migraine drugs. In this systematic review and meta-analysis, we aimed to identify and rate the evidence for efficacy of flunarizine, a repurposed, first- or second-line treatment for migraine prophylaxis.

**Methods:**

A systematic search in MEDLINE, Cochrane CENTRAL, and ClinicalTrials.gov was performed for trials of pharmacological treatment in migraine prophylaxis, following the Preferred Reporting Items for Systematic Reviews (PRISMA). Eligible trials for meta-analysis were randomized, placebo–controlled studies comparing flunarizine with placebo. Outcomes of interest according to the Outcome Set for preventive intervention trials in chronic and episodic migraine (COSMIG) were the proportion of patients reaching a 50% or more reduction in monthly migraine days, the change in monthly migraine days (MMDs), and Adverse Events (AEs) leading to discontinuation.

**Results:**

Five trials were eligible for narrative description and three for data synthesis and analysis. No studies reported the predefined outcomes, but one study assessed the 50% reduction in monthly migraine attacks with flunarizine as compared to placebo showing a benefit from flunarizine with a low or probably low risk of bias. We found that flunarizine may increase the proportion of patients who discontinue due to adverse events compared to placebo (risk difference: 0.02; 95% CI -0.03 to 0.06).

**Conclusions:**

Published flunarizine trials predate the recommended endpoints for evaluating migraine prophylaxis drugs, hence the lack of an adequate assessment for these endpoints. Further, modern-day, large‐scale studies would be valuable in re-evaluating the efficacy of flunarizine for the treatment of migraines, offering additional insights into its potential benefits.

**Supplementary Information:**

The online version contains supplementary material available at 10.1186/s10194-023-01657-3.

## Introduction

Until recently, migraine preventive treatment was limited to a variety of drugs that have been primarily developed to treat other conditions but were later found to be also effective in migraine prevention. Within one of these classes, calcium channel blockers have been studied for the prevention of migraine, of which flunarizine is the most widely used. Flunarizine is a mixed sodium and calcium channel blocker whose preventive effect in migraine might at least in part be attributed to block P/Q-type channels in the brain. The P/Q-type calcium channel is a presynaptic high-voltage-gated calcium channel contributing to vesicle release at synaptic terminals. A number of neurological diseases have been attributed to the malfunctioning of P/Q channels, including migraine.

Calcium channel antagonists prevent calcium from entering cells, resulting in relaxation of heart and vascular smooth muscle, thereby decreasing blood pressure. However, the therapeutic dose of flunarizine was shown to be unlikely to exert calcium-antagonistic effects on cerebral vessels [1]. In contrast, the calcium channel antagonist nimodipine could exert an effect on cerebral arteries at a therapeutic dose, but nimodipine was not better than placebo in migraine prophylaxis [1, 2]. These data suggest that the prophylactic effect of the calcium channel blockers might not be mediated by its direct vasodilatory effect on arteries.

**Fig. 1:**
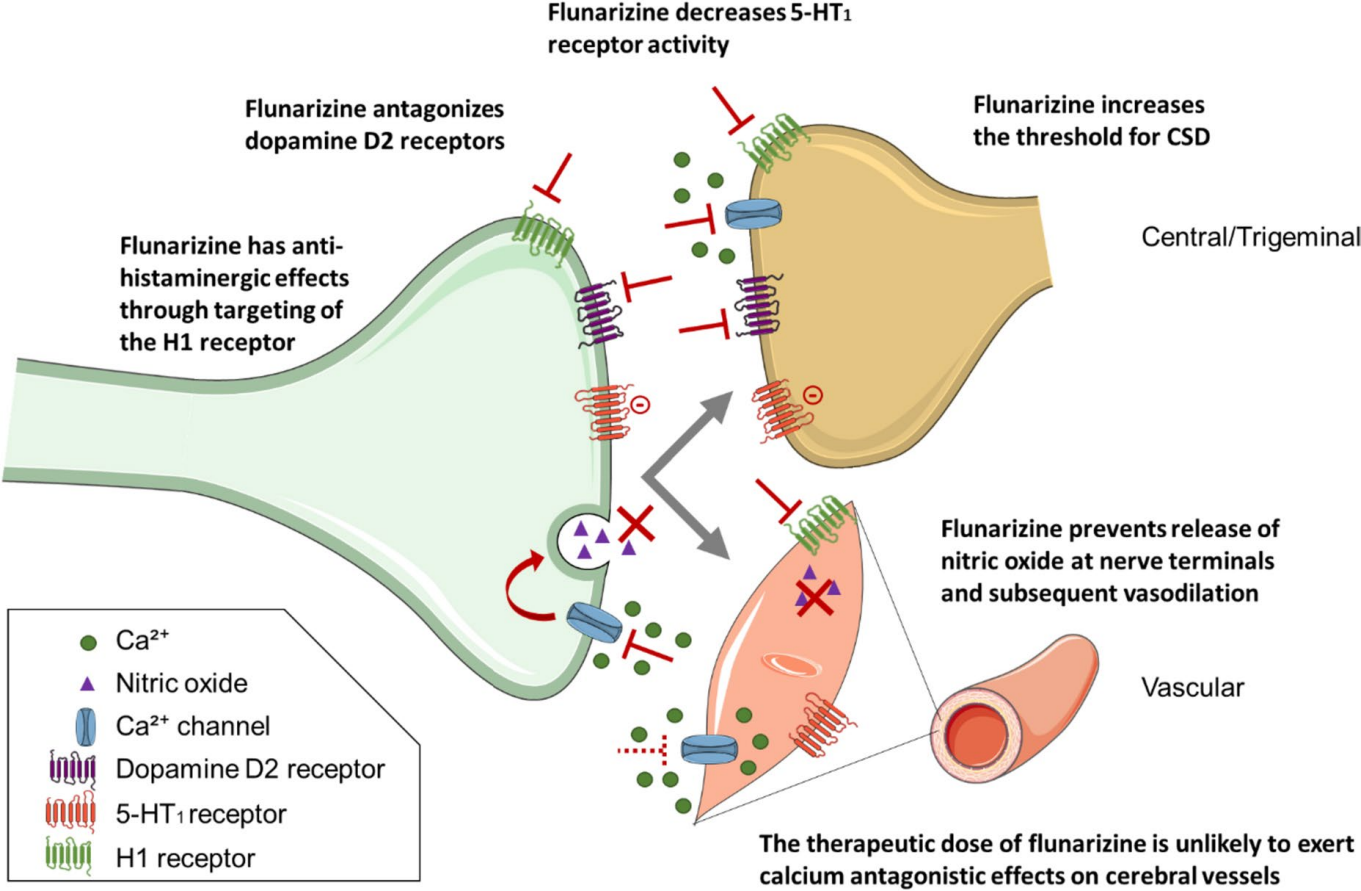
Potential mechanisms of action for the anti-migraine effect of the Ca^2+^ channel blocker flunarizine. Flunarizine affects the production and release of nitric oxide in canine cerebral arteries by blocking the influx of Ca.^2+^ induced by action potentials at nerve terminals [3] and increases the threshold for cortical spreading depression [4, 5]. Moreover, flunarizine antagonizes the dopamine D2 receptor [6], and has antihistaminergic effects through targeting of the H1 receptor [7]. Calcium channel blockers can affect the serotonergic system [8, 9] and prolonged treatment with flunarizine results in decreased activity of 5-HT_1_ receptors in the hippocampus and cerebral cortex of rats [8]. The therapeutic dose of flunarizine for the treatment of migraine is unlikely to exert calcium antagonistic effects on cerebral vessels [1]

Flunarizine was shown to affect the production and release of nitric oxide in canine cerebral arteries by blocking the influx of Ca^2+^ induced by action potentials at nerve terminals [3] and increases the threshold for cortical spreading depression [4, 5], which is thought to underlie migraine aura. Moreover, flunarizine antagonizes the dopamine D_2_ receptor [6], and has antihistaminergic effects, through targeting of the H_1_ receptor, whereas the calcium channel blocker verapamil affects the H_2_ receptor [7]. Another calcium channel blocker—nifedipine -does not target either of these histamine receptors yet it has one clinical study which demonstrated antimigraine effects [8]. Long-term administration of verapamil, flunarizine and nifedipine has also been demonstrated to lead to a reduction in the activity of the 5-HT_1_ receptors in the hippocampus and cerebral cortex of rats [9]. Verapamil, nifedipine and diltiazem inhibit 5-HT_2_ receptors in human brain tissue, while verapamil could also target 5-HT_1A_ receptors, albeit with a lower potency [10]. Given the established efficacy of triptans, which are 5-HT_1_ receptor agonists, in the treatment of migraine, it is worth considering that the anti-migraine effect of calcium channel blockers may be mediated at least in part through their impact on the serotonergic system (Fig. 1).

Flunarizine has been introduced for the management of migraine in the 1980s [11, 12]. Reports on its protective effects against brain hypoxia via the reduction of intracellular calcium overload and inhibitor effects on the contractility of cranial arteries in animal models led to the investigation of its possible prophylactic role in the management of migraine [11, 12]. Flunarizine is suggested in several national treatment guidelines as a drug with level A evidence for migraine prophylaxis, with a recommended dose of 5–10 mg [13]. However, it is important to note that the availability of flunarizine varies among European countries (https://www.ema.europa.eu/en/documents/psusa/flunarizine-list-nationally-authorised-medicinal-products-psusa/00001416/201505_en.pdf) and that it is not marketed in the United States.

The purpose of this study was to re-appraise critically the published trials that evaluated the possible benefits of flunarizine versus placebo in migraine in a systematic approach and illuminate the role of flunarizine on the prophylactic management of migraine through a meta-analysis.

## Methods

This work is the second study of the series aiming to re-appraise different types of classic migraine preventive medications. We conducted a systematic review and meta-analysis and report our results according to the PRISMA statement [14]. We have previously described the methods for this review in detail in the systematic review and meta-analysis focused on the prophylactic role of amitriptyline in migraine [15].

In consultation with a research librarian, we searched MEDLINE, EMBASE, Cochrane CENTRAL, and ClinicalTrials.gov from inception to August 13, 2022 for randomized trials of drug treatments for migraine prophylaxis, without language restrictions (Supplement 1).

Pairs of reviewers, working independently and in duplicate to reduce the potential for errors, screened titles and abstracts of search records and subsequently the full texts of records deemed eligible at the title and abstract screening stage. We included randomized trials that compared flunarizine with placebo for migraine prevention in adults. We excluded trials investigating children or adolescents; or those that randomized a study sample fewer than 25 patients in each treatment arm from data synthesis and analysis [15].

We collected data on trial and patient characteristics (e.g., country of recruitment or severity of migraine), interventions, and outcomes of interest. Our outcomes of interest were pre-specified according to the Outcome Set for preventive intervention trials in chronic and episodic migraine (COSMIG) [16]. We included the proportion of patients with a 50% or more reduction in migraine days per month, change in migraine days per month, and adverse events leading to discontinuation. Monthly headache days or monthly migraine attacks were extracted when monthly migraine days were not reported. We assessed the risk of bias using a modified Cochrane RoB 2.0 tool [17, 18].

For all outcomes, we performed a frequentist random-effects meta-analysis using the restricted maximum likelihood (REML) estimator. We analyzed 50% or more reduction in monthly migraine days as relative risks, monthly migraine days as mean differences, and adverse events leading to discontinuation as risk differences, since we expected many studies to report no or few events with placebo. To facilitate interpretation, we report dichotomous outcomes as number of events per 1,000 patients.

We assessed the certainty of evidence using the GRADE approach [19] and reported using GRADE simple language summaries [15, 20].

## Results

**Fig. 2:**
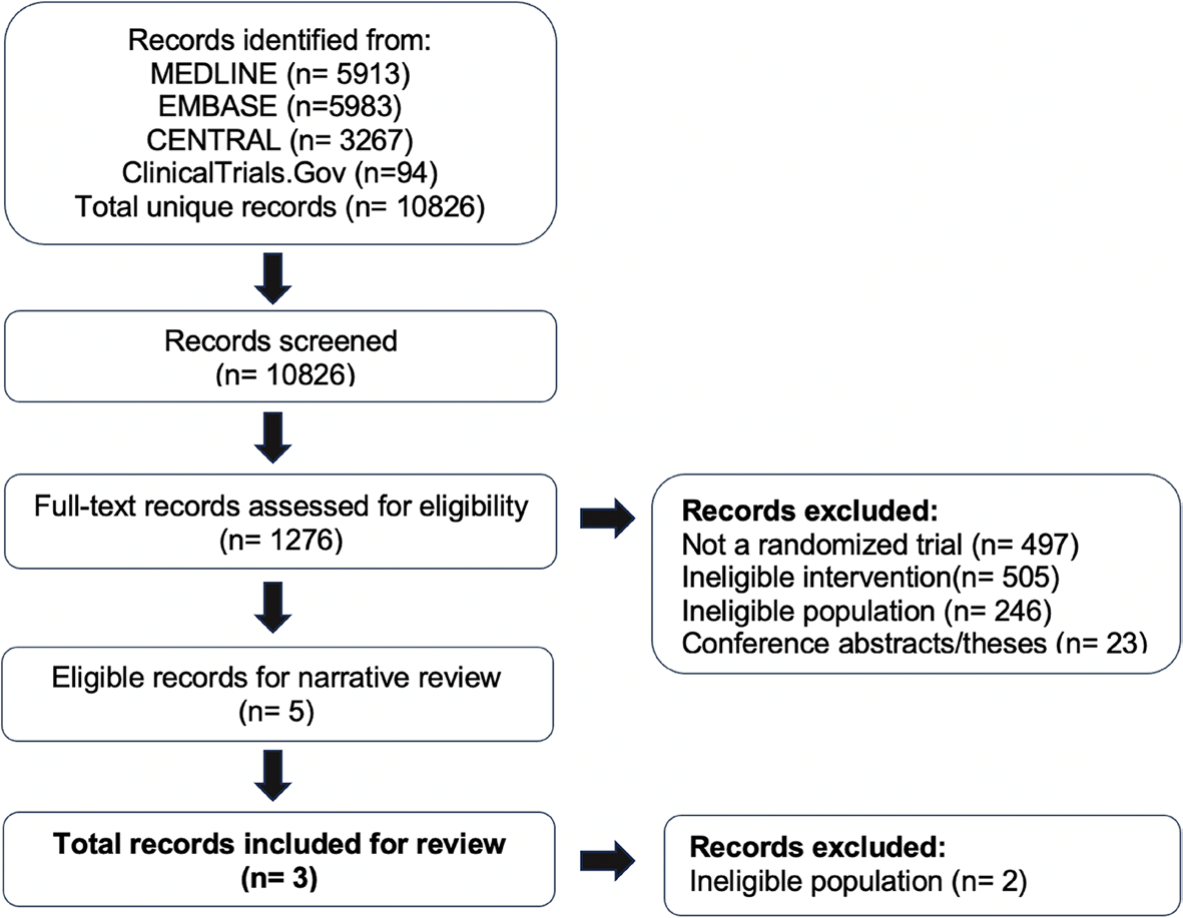
PRISMA flowchart of the study. The systematic search yielded a total of 10,826 unique records. Title and abstract screening resulted in 1276 records potentially eligible, and after full‐text review 5 records proved eligible. Records that did not describe full‐text peer‐reviewed reports of randomized placebo-controlled trials in participants ≥ 18 years old of flunarizine in migraine prevention were excluded

Our systematic literature review yielded 10,826 records, 1276 records proved to be potentially eligible after title and abstract screening, of which five trials were eligible for the narrative description [21–25] and three for data synthesis and analysis [21, 24, 25]. Details of the study selection are shown on the PRISMA flowchart diagram (Fig. 2).

### Narrative description of flunarizine in placebo-controlled trials

The first conducted study [21] published in 1981 reported a median attack frequency reduction of 57% in 29 patients treated with flunarizine 10 mg/day versus 14% in 29 patients treated with placebo. In this study, the duration and severity of attacks did not change; mild sedation and dry mouth were reported as main adverse events (AEs). In a second study flunarizine was judged beneficial in 59% of a sample of 17 participants treated with flunarizine 10 mg/day compared to 18 treated with placebo, with a mean monthly attack reduction at month three of -2.5 in the flunarizine group versus -1.2 in the placebo group. Additionally, flunarizine was associated with sedation at the beginning of the treatment [22]. A year later a small cross-over study (*n* = 9) reported a ~ 50% reduction of migraine attacks with flunarizine 10 mg/day in the third month, interestingly no AEs were reported [23]. In another cross-over placebo-controlled study including 29 patients with a duration of 16 weeks for each treatment period and a 4-week baseline/wash-out period [24], the authors reported that the median number of migraine attacks measured per four-week period was 3.5 during baseline/washout period, 3.2 during placebo treatment, and 2.0 while on flunarizine. In this study, migraine attack frequency, hours with migraine, and migraine index were statistically significantly reduced by 50% or more in the last four-week period on flunarizine. In the last referred study, including 50 patients treated with flunarizine, flunarizine showed a statistically significant 50% reduction in attack frequency compared to 40% in the placebo group [25].

Overall, the design of these older studies is not in line with the current international guidelines for controlled drug trials in migraine, which were first published after 1991 [26]. The definition of migraine and the criteria used for its classification varied between the referred trials. Headaches were classified as common migraine or classical migraine based on the Ad Hoc Committee on Classification of Headache of the NIH [27] in three trials [21–23] and the modified criteria of the Ad Hoc Committee on the Classification of Headache by Olesen et al. [28] in another one [24]. One trial performed in 1991 [25] used the first edition of the International Headache Classification (ICHD-1) [29] and recruited migraine individuals with or without aura (Table 1).

**Table 1:** Placebo controlled randomized clinical trials of flunarizine. trial characteristics

***Study name***	***Study design***	***Funding***	***Country***	***Sample size***	***Age, median (range)***	***% Male***	***Types of migraine***	***Drug dosage (daily)***	***Active Tx period (weeks)***	***Outcome measures***	***Tolerability***
^d^**Louis 1981 **[21]	PCS	NR	Belgium	58 (29 in each arm)	29 (20–47)	50	Common migraine (31%) or Classical migraine (69%)^a^	10 mg	12	Reduction in: • Monthly migraine attacks (at least one attack at 3^rd^ mo: 17.2% vs 55.2%, *p* = 0.006) • Duration and severity of migraine attacks, did not differ between two interventions	Mild day-time sedation in 2 patients on flunarizine (without withdrawal) Dry mouth; in 2 controls (without withdrawal)
^d^**S**ø**rensen 1986 **[24]	PCS, Cross-over	NR	Denmark	29^e^	40 (19–63)	20.7	Common migraine^b^	10 mg	16	Reduction in: • Monthly migraine attacks (reduction in number of attacks at 4^th^ mo: 50% vs 15%, *p* = 0.02) • Duration and severity of migraine attacks (p = NS, for both)	Mild day-time sedation in 3 patients on flunarizine (1 withdrawal)
^d^**Freitag 1991 **[25]	PCS	NR	US	101 (50 in flunarizine arm)	NR	25.7	MwA (32.7%) or MwoA (67.3%)^c^	10 mg	16	Reduction in: • Number of migraine attacks from baseline to the end (50% vs 39.9%, *p* = 0.018) • Duration and severity of migraine attacks (reduced in flunarizine arm, but *p* = NS for both)	5 withdrawals among patients on flunarizine^††^ 3 withdrawals among controls
**Frenken 1984 **[22]	PCS	NR	Netherlands	35 (17 in flunarizine arm)	NR	17.1	Common migraine (60%) or Classical migraine (40%)^a^	10 mg	12	Reduction in: • Monthly migraine attacks (significant reduction in flunarizine arm; *p* = 0.029 at 1^st^ mo) • Duration of migraine attacks (p value is not reported) • Severity of migraine attacks (*p* = NS)	Day-time sedation in 7, weight gain in 3 and other AEs in 2 patients on flunarizine Day-time sedation in 3, stomach complaints in 4, and other AEs in 1 of controls
**Mendenopoulos 1985 **[23]	PCS	NR	Greece	20 (9 in flunarizine arm)	20–65	20	Classical migraine ^a^	10 mg	12–16	Reduction in: • Monthly Migraine attacks (50% reduction in attacks after 3 mo; *p* = 0.033) • Duration and severity of migraine attacks (significant reduction in flunarizine arm; *p* = 0.037 and *p* = 0.006, respectively, after 3rd month)	No AEs

*AEs* Adverse events, *FU* Follow-up, *MwA* Migraine with aura, *MwA* Migraine without aura, *NR* Not reported, *NS* not significant, *PCS* Prospective cohort study, *Tx* Treatment

^a^defined according to the Ad Hoc Committee on Classification of Headache of the NIH [25]

^b^defined according to the modified criteria of the Ad Hoc Committee on the Classification of Headache by Olesen et al. [26]

^c^defined according to the first edition of the International Headache Classification [27]

^d^Studies included in the meta-analysis

^e^One patient withdrew on placebo because of increasing number of migraine attacks

^f^Details of the adverse events leading to withrawal were not given in the article

Three trials [21, 24, 25] randomized more than 25 patients in each treatment arm and only one of them had a larger sample size of overall 101 patients [25]. Age groups were not reported in two records [22, 25], middle-aged patients were mostly reported to be recruited in the remaining trials [21, 23, 24]. Most of the patients were females. None of the RCTs comparing flunarizine versus placebo reported information on funding to support the study. Research methods were overall not described in detail. Patients who were assigned following the withdrawal of anti-migraine medications [21–23, 25] or those taking drugs for migraine prevention were excluded [24]. However, the baseline medication-free period was reported in only one record [23]. Only two trials stated that patients were asked to complete a diary documenting headache frequency, characteristics and AEs [21, 24].

All RCTs reported a significant reduction in frequency of migraine attacks for flunarizine 10 mg/day over placebo. The duration and severity of migraine attacks were also assessed and tended to decrease in the flunarizine-treated patients in comparison with the placebo-treated ones in only two trials [23, 25] reaching statistical significance in one of these trials [23].

#### Data synthesis and analysis

**Fig. 3:**
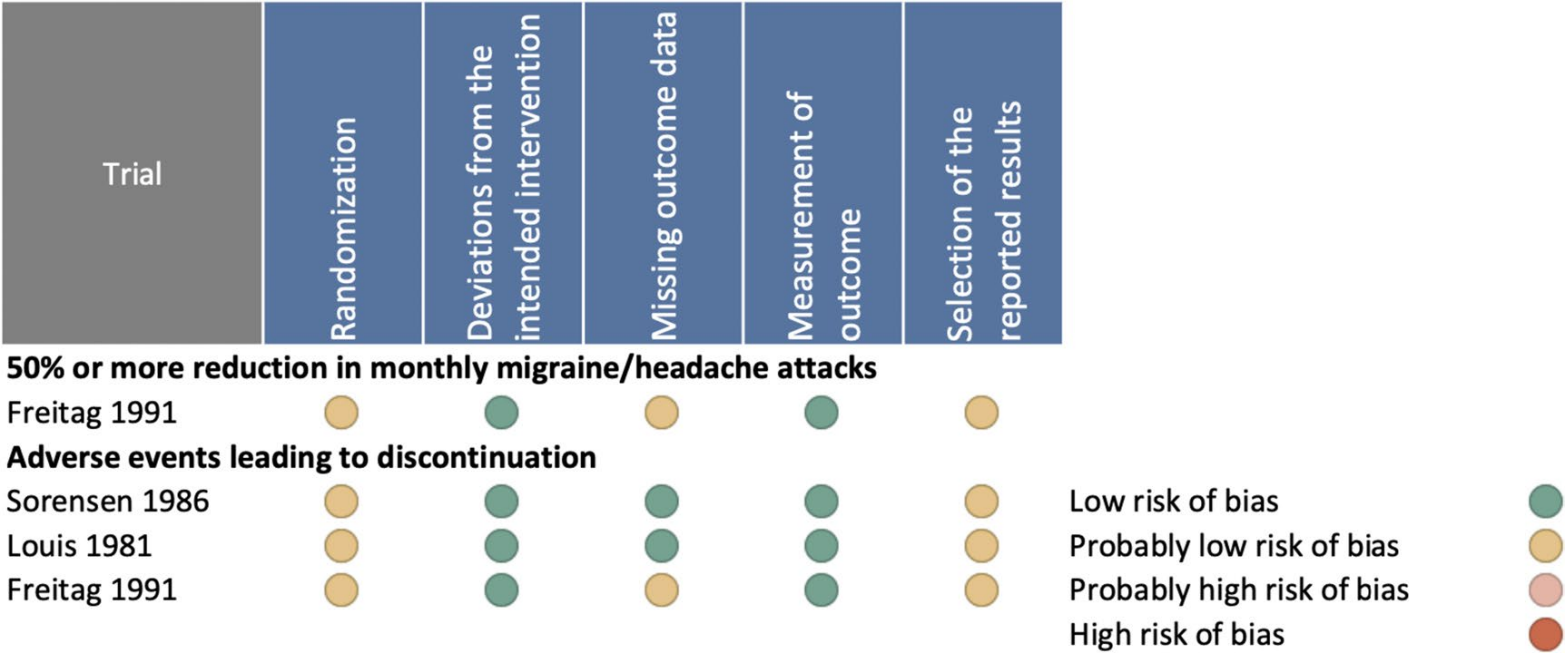
Risk of bias assessment

Three trials with a total of 188 participants were eligible and included for the quantitative analysis [21, 24, 25]. The other two trials were excluded since they assigned less than 25 individuals in each group [22, 23]. Two of the included trials were performed in Europe [21, 24] and one in the USA [25]. Characteristics of the five trials, eligible for narrative description, including study design, sample size, flunarizine dosage, period of active treatment, median age, sex distribution, type of migraine, outcome measures, and tolerability issues are presented in Table 1, while the risk of bias of eligible trials is presented in Fig. 3.

#### 50% responder rate

The outcome of 50% or more reduction in migraine days per month was not reported. One study reported on the 50% reduction in migraine attacks in favor of flunarizine with a low or probably low risk of bias [25] (Fig. 3).

#### Monthly migraine days

No available data.

#### Adverse events leading to discontinuation

**Fig. 4:**
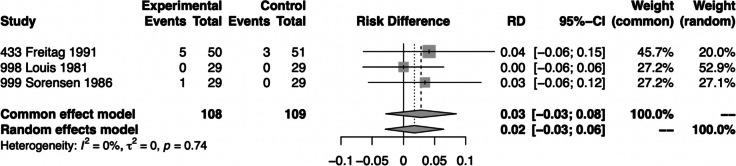
Forest plot of analysis comparing flunarizine with placebo for adverse events leading to discontinuation

We could only perform a quantitative analysis on AEs leading to discontinuation showing that significantly more participants treated with flunarizine discontinued treatment than those treated with placebo (Fig. 4). In the pooled analysis, ten participants treated with flunarizine reported AEs but six withdrew from the treatment [21, 24, 25]. In the placebo arm, five participants reported AEs and three withdrew. This outcome was rated as low or probably low risk of bias for all three RCTs and of high certainty according to the GRADE approach (Fig. 3, Table 2). Mild daytime sedation and weight gain were the most common AEs leading to discontinuation [21, 22, 24, 25]. However, several AEs such as dry mouth and stomach complaints as well as daytime sedation were also reported by patients treated with placebo [21, 22].

**Table 2:** Flunarizine compared to placebo for migraine prophylaxis

Patient or population: migraine Intervention: prophylaxis with flunarizine Comparison: placebo					
**Outcomes**	**№ of participants** **(studies)** **Follow-up**	**Certainty of the evidence** **(GRADE)**	**Relative effect** **(95% CI)**	**Anticipated absolute effects**	
				**Risk with placebo**	**Risk difference with flunarizine**
50% or more reduction in monthly migraine days	No data				
Monthly migraine days	No data				
Adverse events leading to discontinuation	188 (3 RCTs)	High	RD 0.02 (-0.03 to 0.06)	0 per 1,000	20 more per 1,000 (30 fewer to 60 more)

*CI* Confidence interval, *RD* Risk difference

GRADE Working Group grades of evidence: High certainty; we are very confident that the true effect lies close to that of the estimate of the effect

The risk in the intervention group (and its 95% confidence interval) is based on the assumed risk in the comparison group and the relative effect of the intervention (and its 95% CI)

## Discussion

Flunarizine is one of the first oral, repurposed drugs used for migraine prevention. Although RCTs comparing flunarizine with placebo share several important methodological limitations, flunarizine still stands as a drug of choice in Europe, with class A evidence [30]. That is the reason why flunarizine is among the list of first-line treatments that migraine patients must fail to be eligible for specific novel migraine preventives in many countries. The present study revealed that the evidence on this medication efficacy is limited to studies mostly conducted more than a quarter of a century ago and empiric knowledge in clinical practice. Available trial data are not of enough quality to recommend its use as a migraine preventive treatment with class A level of evidence.

Although our findings are generally in line with a recently published comprehensive meta-analysis [31], with different methodological approaches though, we are coming in contrast in supporting the attribution of a Level A evidence for efficacy of flunarizine, on which also the authors raise concerns on the quality of data from older studies. The results of trials done in the past following different quality criteria should be evaluated with modern quality criteria data and scored. Currently, a grade A recommendation should be a result of large, RCTs showing consistent, impressive benefits with few AEs and minimal inconvenience and cost [19].

Our aim was to explore the existing evidence following the best methodological boundaries, rather than to reach clinically relevant conclusions by applying sensitivity analyses and risk of bias assessments. Our search concluded that the existing evidence for the efficacy of flunarizine in migraine prevention is of very low quality, in contrast to modern preventives, which are additionally disease-specific and mechanism-based.

So far, all five trials yielded by our systematic search showed a significant reduction in migraine attacks over placebo in the limited observational period that each study was conducted. Current international guidelines on migraine prevention trials recommend as primary outcome the change of migraine days from baseline and, alternatively, the change from baseline in moderate/severe headache days or 50% responder rate for the reduction of migraine days [32]. The outcome measured in the referred trials included reduction in monthly migraine attacks, duration, and severity of migraine attacks. Therefore, the recommended outcome measures [32] could not be analyzed in this reappraisal study. Additionally, the study populations in all trials were very small, which raises questions regarding the strength of study results. Available studies had an additional number of limitations [21–25]: a) none of the studies mentioned sample size calculation, b) lack of detailed methodology description (inclusion and exclusion criteria, definition of migraine with or without aura, statistical analysis plan, and randomization methods), c) active treatment periods were short and varied between 12–16 weeks, d) details regarding baseline observation period were lacking, e) blinding procedure was not described in most of them, f) drop-out rates and reasons, except withdrawals due to AEs were not clearly stated, g) comorbidity of other primary headaches.

We could only analyze the AEs leading to discontinuation. Day-time sedation and weight gain were the most commonly reported AEs of flunarizine. We found a significant difference between placebo and 10 mg flunarizine, regarding comparison of AEs leading to discontinuation, which is in line with experts’ clinical experience. Accordingly, flunarizine seems to be a safe and well-tolerated drug. On the other hand, our findings should be interpreted cautiously, as I^2^ value had an absolute homogeneity (I^2^ = 0%) (Fig. 4), which may also be criticized to show the low validity of this latter finding. Additionally, one study reports no AEs in total [23], which could be due to poor and incomplete recording during the trial and/or the small numbers of participants (*n* = 9).

According to the GRADE approach the certainty of evidence is rated high for the outcome of AEs leading to discontinuation (Fig. 4). As mentioned, there is a noted mismatch in the design of these older studies compared to current international guidelines for controlled drug trials in migraine. There is also a variance in the migraine definition and classification criteria used among the flunarizine trials. This issue is classified as "indirectness", which pertains to the difference between the queries addressed in individual studies and the question that the systematic review aims to answer.

As per the GRADE guidance, we could downgrade the rating for indirectness if the effect reported in the studies did not accurately represent the effect concerning our question. Despite these issues, we did not downgrade the rating since the safety and tolerability of drugs is unlikely to be affected by diagnostic criteria. Nevertheless, we considered downgrading the certainty of evidence to moderate due to imprecision, based on the Minimum Important Difference (MID). Since the effect estimate meets the MID, we are rating the certainty that flunarizine increases the proportion of patients that experience AEs leading to discontinuation. Nevertheless, the confidence interval crosses the MID, which is indicative for imprecision. Hence, we conclude that flunarizine probably increases the proportion of patients that experience AEs leading to discontinuation.

Furthermore, results from post-marketing cohort studies regarding discontinuation due to AEs are provided. One study including 838 participants (aged 7–93 years) treated with flunarizine 5–20 mg (> 60% in 10 mg flunarizine group) up to 8 months, reports a percentage of 6% of subjects who discontinued due to AEs [33]. In another study, which was published in a Journal supplement only 14 out of 1435 participants, treated up to 6 months, with a follow-up period of an additional 6 months, discontinued due to AEs [34]. Overall, the most common adverse events reported were weight gain, fatigue and drowsiness.

Two head-to-head studies comparing flunarizine with propranolol showed an almost equal efficacy and tolerability profile [35, 36]. One study of 808 subjects treated for 16 weeks reported that flunarizine 10 mg was at least as effective as 160 mg propranolol concerning all evaluated parameters, and both drugs were well tolerated [35]. Another double-blind study comparing flunarizine 10 mg and propranolol 160 mg in more than 400 patients diagnosed with “classical migraine”, reported similar efficacy results (number, duration, and severity of attacks) [36]. Sedation or fatigue, gastric pain, vertigo and nausea were reported as the most important AEs in both groups [36].

Flunarizine could be beneficial as a treatment choice in relation to specific comorbidities. Considering its inhibitory effects on the calcium-related contraction of vascular smooth muscle, flunarizine may be a good option for prophylaxis in migraine individuals with cardiovascular diseases such as arrythmias [37]. Additionally, the vestibular depressive effect of this drug was also demonstrated in animals and humans [11], and recent trials reported the benefit of flunarizine on vestibular symptoms in patients with vestibular migraine [38, 39].

## Conclusions

Five trials of flunarizine as prophylactic agent yielded by our systematic search showed a significant reduction in migraine attacks over placebo in limited observational periods, in small population groups and selected outcome parameters. Based on these results, flunarizine has been used as a first or second line prophylactic treatment for migraine in many countries. The present critical and systematic reappraisal of these trials, in accordance with current international guidelines for controlled trials on migraine prevention reveals an insufficiency of data to support the attribution of a Level A evidence for efficacy. The introduction of novel migraine-specific drugs with good evidence of efficacy and excellent tolerability challenges the role of traditional oral preventatives such as flunarizine. Further large-scale studies, head-to-head trials or real-world evidence would be useful to support the role and efficacy of flunarizine in migraine prevention.

### Supplementary Information

**Additional file 1. **

## Abbreviations

Aes: Adverse events
Ca^2+^: Calcium cations
CGRP: Calcitonin gene-related peptide
FDA: Food and Drug Administration
COSMIG: Outcome Set for preventive intervention trials in chronic and episodic migraine
CSD: Cortical spreading depression
PRISMA: Preferred Reporting Items for Systematic Reviews
MMDs: Monthly migraine days
RCT: Randomised Controlled Trial
